# Intra-osseous injection of donor mesenchymal stem cell (MSC) into the bone marrow in living donor kidney transplantation; a pilot study

**DOI:** 10.1186/1479-5876-11-96

**Published:** 2013-04-11

**Authors:** Hyunah Lee, Jae Berm Park, Sanghoon Lee, Soyoung Baek, HyunSoo Kim, Sung Joo Kim

**Affiliations:** 1 Office of Biomedical Professors, Samsung Medical Center, Sungkyunkwan University School of Medicine, Seoul, Korea; 2 Department of Surgery, Samsung Medical Center, Sungkyunkwan University School of Medicine, Seoul, Korea; 3 FCB-Pharmicell Co.Ltd, Seoul, Korea

**Keywords:** Donor MSC, Intra-osseous injection, Living Donor Kidney Transplantation (LDKT), Immune response

## Abstract

**Background:**

Mesenchymal stem cells (MSCs) are multi-potent non-hematopoietic progenitor cells possessing an immune-regulatory function, with suppression of proliferation of activated lymphocytes. In this study, adult living donor kidney transplantation (LDKT) recipients were given MSCs derived from the donor bone marrow to evaluate the safety and the feasibility of immunological changes related to the intra-osseous injection of MSC into the bone marrow.

**Methods:**

MSCs were derived from negative HLA cross-match donors. Donor bone marrow was harvested 5 weeks prior to KT. At the time of transplantation, 1 x 10^6^ cell/kg of donor MSC was directly injected into the bone marrow of the recipient’s right iliac bone. Patients’ clinical outcomes, presence of mixed chimerism by short tandem repeat polymerase chain reaction, analysis of plasma FoxP3 mRNA and cytokine level, and mixed lymphocyte reaction (MLR) were performed.

**Results:**

Seven patients enrolled in this study and received donor MSC injections simultaneously with LDKT. The median age of recipients was 36 years (32 ~ 48). The number of HLA mismatches was 3 or less in 5 and more than 3 in 2. No local complications or adverse events such as hypersensitivity occurred during or after the injection of donor MSC. There was no graft failure, but the biopsy-proven acute rejections were observed in 3 recipients during the follow-up period controlled well with steroid pulse therapy (SPT). The last serum creatinine was a median of 1.23 mg/dL (0.83 ~ 2.07). Mixed chimerism was not detected in the peripheral blood of the recipients at 1 and 8 week of post-transplantation. Donor-specific lymphocyte or T cell proliferation and Treg priming responses were observed in some patients. Plasma level of IL-10, a known mediator of MSC-induced immune suppression, increased in the patients with Treg induction.

**Conclusion:**

Donor MSC injection into the iliac bone at the time of KT was feasible and safe. A possible correlation was observed between the induction of inhibitory immune responses and the clinical outcome in the MSC-kidney transplanted patients. Further research will be performed to evaluate the efficacy of MSC injection for the induction of mixed chimerism and subsequent immune tolerance in KT.

## Background

Although kidney transplantation (KT) is a widely accepted and effective treatment option for patients with end stage renal disease, the need for life-long immunosuppression puts the patient at constant risk. The risk of opportunistic infections, drug toxicity, and malignancies is increased in these patients [1]. Moreover, even with immunosuppression not all patients can evade the occurrence of acute and chronic rejection. Over several decades many immunosuppressive agents and regimens have been introduced and the incidence of acute rejection in the early period after the transplantation has decreased. However most immune suppressive regimens have limitations in long-term prevention of progressive chronic allograft nephropathy after the transplantation, ultimately leading to graft failure [1-3].

Mesenchymal stem cells (MSCs) are multi-potent non-hematopoietic progenitors found in the bone marrow, cord blood, heart, skeletal muscle, and adipose tissue. MSCs differentiate *in vitro* and *in vivo* into tissues of mesenchymal origin [4-6]. MSCs provide support for the growth and differentiation of hematopoietic progenitor cells in bone marrow micro-environments. MSCs also have immune regulatory effect by suppressing activated lymphocytes proliferation and DC function [7-9].

The immune regulatory function of MSCs has been studied in the field of hematopoietic stem cell transplantation primarily for the treatment of severe, steroid-resistant graft-versus-host disease [10-12]. Neither acute nor long-term adverse events have been reported after infusion of MSCs.

There is very recent research on the immune-regulatory role of MSCs in solid-organ transplantation [13,14]. Tan and others [14] published randomized controlled trial results with autologous MSC in kidney transplants. After the living-related donor kidney transplantation, patients were treated with conventional immune suppression regimen (steroids, mycophenolate, mofetil, and either cyclosporine or tacrolimus) with autologous MSCs or anti-IL2 receptor antibody. The authors found a beneficial effect of MSCs over anti-IL2 receptor antibody induction therapy, resulting in lower incidence of acute rejection and opportunistic infection.

With a protocol differing considerably from Tan’s, in the present study the effect of donor-derived MSCs on the recipients of living donor KT (LDKT) was observed. The most significant difference of present study from Tans was the route and the timing of MSC administration. MSC introduced into the bone marrow of iliac bone near the kidney graft at the time of transplantation. Simultaneous intra-osseous injection of MSC with kidney transplantation could reduce the patient’s discomfort with invasive way of cell administration. In this study, the feasibility, tolerability and the possibility of regulatory immune induction with intra-osseous injection of donor MSC in LDKT was observed**.**The significance of MSCs on the reduction of conventional immune-suppression regimen with induction of transplanted organ-specific immune-tolerance will be discussed.

## Materials and methods

### Patients’ characteristics and criteria

Seven patients were enrolled in this study during August through November in 2007. The clinical protocol of this study has been approved by Institutional Review Board of Samsung Medical Center (SMC 2006-06-031-001) and Korea Food and Drug Administration (KFDA) (KFDA-BM-350, 20070125). Patients between age 20 and 65 years receiving living donor KT for the first time were eligible for the study. Patients were excluded if they showed positive in HLA-cross-match, PRA > 30%, and positive serologic markers for hepatitis B, hepatitis C or HIV. Patients were informed of the investigative nature of this study, and written consent in accordance with institutional regulations was obtained prior to study entry. Median age was 36 years (range 32–48) and the characteristics of the patients enrolled in the study are detailed in Table 1.

**Table 1:** Characteristics of the patients

		**MSC group (n = 7)**
**Recipient**	**Age (year)**	**36 (32 ~ 48)**
	**Sex (M : F)**	**3 : 4**
	**Etiology**	
	**Unknown**	**4**
	**Diabetes**	**0**
	**Hypertension**	**0**
	**IgA nephropathy**	**2**
	**GN**	**1**
	**Underlying DM / HTN**	**2 / 3**
**Donor**	**Age (year)**	**38 (29 ~ 48)**
	**Sex (M:F)**	**6 : 1**
	**Relation (Related /Unrelated)**	**7 : 0**
	**HLA mismatch (0/1/2/3/4)**	**1 / 0/ 3 / 1 / 2**
	**DR mismtach (0/1 /2)**	**2 /5 /0**

GN, glomerulonephritis, DM, diabetes mellitus; HTN, hypertension.

### MSC culture and infusion

All the manufacturing and product testing procedures for the generation of clinical-grade MSCs were performed under good manufacturing practice (GMP) conditions (Pharmicell Co Ltd, Seongnam, South Korea) according to the Korea Food and Drug Administration (KFDA) approved-protocols (KFDA-BM-350, 20070125). MSCs were derived from negative HLA-cross-match donors. Five weeks prior to the transplantation, approximately 50 mL of bone marrow was obtained from the posterior superior iliac crest of donor. Mononuclear cells were separated from the bone marrow by density gradient centrifugation (Ficoll-Paque, 1.077 g/L, Sigma, St. Louis, MO) and washed with phosphate-buffered saline (PBS). Cells were resuspended in Dulbecco modified Eagle medium-low glucose (DMEM; Gibco, Grand Island, NY) containing 10% fatal bovine serum and 100 U/mL penicillin/100 μg/mL streptomycin (Gibco), and plated at a density of 1.0-1.5 × 10^5^ cells/cm^2^ in 75 cm^2^ or 175 cm^2^ flasks. Cultures were maintained at 37°C in a humidified atmosphere containing 5% CO_2_. After 5–7 days, non-adherent cells were removed by replacing the medium and the adherent cells were cultured another 2–3 days. After reaching 70-80% confluency, the adherent cells were detached with Trypsin containing EDTA (Gibco) and re-plated at a density of 4–5 × 10^3^ cells/cm^2^ in 175 cm^2^ flasks. The cells were serially sub-cultured up to passage five for infusion. On the day of injection, MSCs were harvested using Trypsin, washed twice with PBS and once with saline solution, and re-suspended to a final concentration of 5.0 × 10^6^ cells/ mL in saline solution. Criteria for release of MSCs for clinical use included absence of microbial contamination (bacteria, fungus, virus and mycoplasma) and viability greater than 80% when assessed using a trypan blue exclusion assay. Also clinical grade MSCs were characterized by expression of CD73 and CD105 (more than 90%) and absence of CD14, CD34, and CD45 (less than 3%) (data not shown). Infusion of MSCs (1 × 10^6^ cell/kg) directly into the bone marrow of the recipient’s iliac bone was done simultaneously with transplantation of the kidney. Changes in the patient’s vital signs or appearance of the skin, signs of anaphylaxis were continuously monitored during and after the injection of MSCs.

### Immune suppression

Patients with MSC-kidney transplants were treated with conventional immune-suppression regimen. Starting from the day of KT, patients were given anti-thymocyte globulin for induction, for a total of 8–10 days (1.5 mg/kg daily). The maintenance immune-suppression regimen consisted of a calcineurin inhibitor, mycophenolate mofetil (MMF) and steroids. Dosage was decreased at the clinician’s discretion when such adverse events as leukopenia or severe diarrhea occurred.

### Clinical evaluation

Patients were monitored throughout the study by clinical examination and toxicity assessment. Hematology, blood chemistry, urinalysis, immune-suppressive drug levels (cyclosporine or tacrolimus), CMV antigenemia, polyoma and parvovirus PCR tests were done according to protocol schedule. Ultrasonography-guided percutaneous core needle biopsy of the transplanted kidney was done at 12 months post-transplant.

### Diagnosis and treatment of acute cellular rejection (ACR)

Upon periodic assessment of serum creatinine level, a 20% or higher increase raised clinical suspicion of ACR. Diagnosis of ACR was done via percutaneous needle biopsy of the transplanted kidney, with pathologic evaluation according to the Banff criteria. Once a diagnosis of ACR was made, the patient was put on steroid pulse therapy (SPT, solumedrol 500 mg/day for 3 days), then tapering back to the dose prior to therapy. If the rejection episode was steroid-resistant, second-line agents such as thymoglobulin were used.

### Chimerism analysis

Short tandem repeat polymerase chain reaction (STR-PCR) was used to identify the presence of mixed chimerism in the recipient’s peripheral blood on day 7, 60, and 180 days following transplantation.

### FoxP3 Quantitation using real time PCR

Experiment performed by following the manufacturer providing procedures. Patients urine was collected on day 0, before the transplantation and various time points (days 7, 30, 90, 180 and 365) after the transplantation. Total RNA was extracted from recipient urine pellets by RNeasy minikit (Qiagne, Chatsworth, CA) and 1 μg was reverse-transcribed using Superscript III (Invitrogen, Carlsbad, CA). PCR reaction mixtures contained universal mastermix (Applied Biosystems, Foster City CA), cDNA, gene-specific primers and probes. Primers were purchased from Sigma Genosys (Woodlands, TX). 18S ribosomal RNA (rRNA) levels were analyzed as housekeeping gene expression for relative quantification. The relative expression of FoxP3 to 18S rRNA was expressed as ΔCt values as discussed by Livak et al. [15].

### Mixed lymphocyte reaction

A heparinized blood sample was collected from the recipient before (day-35) and after (day +30) the LDKT. Separated recipient lymphocytes were seeded triplicates in 96-well plates (2 × 105 cells/100 μl/well) with irradiated donor lymphocytes (1 × 105 cells/100 μl/well) with or without ConA (1 μg/ml final concentration) for the culture at 37°C, in humidified and 5% CO2-conditioned air for 4 days. Cultures were loaded with 1 μCi ^3^H-thymidine/well (Perkin-Elmer Inc., MA, USA) during the last 18 h. Cells were harvested on the glass micro-fiber filter using a PhD® cell harvester (Cambridge Technology Inc., Cambridge, MA, USA). The proliferative response was determined by ^3^H-thymidine incorporation using a liquid scintillation counter (Beckman LS 6500; Beckman Instruments Inc., Fullerton, CA, USA).

### Measurement of cytokine level

Alteration in blood circulating concentrations of IL-10, IL-6, and IFN-γ was measured by ELISA. Plasma was obtained from the patient’s peripheral blood before, and various time points (35 days before and 4, 7, 30, 90, 180 days after the transplantation) and stored at −70°C until the ELISA was performed using commercial kit from OptEIA™ (e-bioscience, San Jose, CA, USA).

### Statistical analysis

For analysis of the immune response, values before and after transplantation in the patients were used and compared by Student’s t-test. A value of *P* < 0.05 was considered statistically significant.

## Results

### Clinical outcome

Seven patients received donor MSC injection simultaneously with LDKT (intra-osseous injection). No adverse events like hypersensitivity occurred during or after the injection of donor MSC. Also there were no local complications related to the intra-osseous injection of donor MSC into the right iliac bone of the kidney recipient.

In the MSC-treated group, acute rejection was observed during the 12 month follow-up period in three patients (Table 2). Acute antibody-mediated rejection occurred at day 9 after the transplantation in patient 2 which was treated by IVIG and plasmapheresis. In Patient 7, acute cellular rejection was observed at 43 days and 613 days of post-transplantation, controlled well with steroid pulse therapy (SPT). Another case of acute cellular rejection was detected on the protocol biopsy at 12 months after the transplantation in patient 4 which caused of SPT. Two cases of borderline change observed in the patients 1 and 6 were not associated with clinical signs of rejection and not require any additional treatment (Table 2). The last serum creatinine of the 7 recipients was a median of 1.23 mg/dL (0.83 ~ 2.07) and no graft failure was found (data not shown). Characteristics and clinical rejection phenomenon of four patients in non-MSC control group were similar to MSC-treated group (Additional file 1: Tables S1 and S2)

**Table 2:** Profiles of the kidney transplantation recipients

**MSC group (n = 7)**							
**No**	**Age/Sex**	**Etiology**	**Donor**		**HLA mismatch**	**Rejection within 12Mo**	**Protocol Bx at 12 Mo**
			**Relation**	**Age/Sex**			
1	34/M	unknown	Brother	32/M	3	-	Borderline change
2	48/F	unknown	Brother	40/M	2	AMR	No AR
3	40/M	unknown	Brother	48/M	0	-	No AR
4	32/F	CGN	Brother	29/M	2	-	ACR
5	36/M	IgA nephropathy	Sister	38/F	2	-	No AR
6	36/F	unknown	Brother	33/M	4	-	Borderline change
7	40/F	IgA nephropathy	Brother	38/M	4	ACR	ACR

CGN, chronic glomerulonephritis; AR, acute rejection; AMR, antibody-mediated rejection; ACR, acute cellular rejection; HTN, hypertension.

### Chimerism induction

Chimerism analysis was done on day 7, 2 months and 6 months post-transplantation. Mixed chimerism was not detected in the peripheral blood of the patients at any time point. (Data not shown.)

### FoxP3 mRNA levels

**Figure 1:**
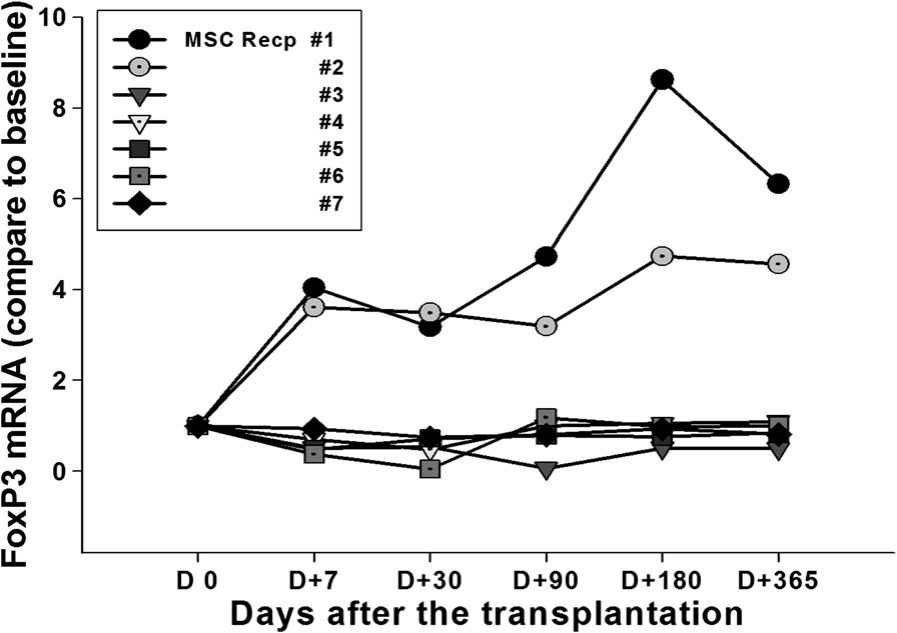
**Level of FoxP3 mRNA.** Quantitative real time PCR with recipient urine pellets was performed and analyzed FoxP3 mRNA expression to estimate the induction of regulatory T cells. Following the methods in the Materials & Methods section, Foxp3 mRNA level in the MSC-kidney transplantation patients was measured. Patients’ urine was collected from day 0 and days 7, 30, 90, 180 and 365 after the transplantation. Foxp3 mRNA level was compared to baseline level (= 1).

Quantitative real time PCR was performed and analyzed for FoxP3 mRNA expression to estimate the induction of regulatory T cells. In the MSC treated patients 1 and 2, long-lasting induction of FoxP3 level was observed (Figure 1). Foxp3 mRNA level in the rest of the MSC group or non-MSC group patients was not significantly altered from the baseline measured with pre-transplantation sample obtained on day 0 (Figure 1 & Additional file 2: Figure S1). Overall changes at each time point were compared between non-MSC and MSC group (Additional file 3: Figure S2). Different from non-MSC control group, tendency of continuous increase of FoxP3 mRNA level was observed in MSC-treated.

### Donor-specific lymphocyte proliferation

**Figure 2:**
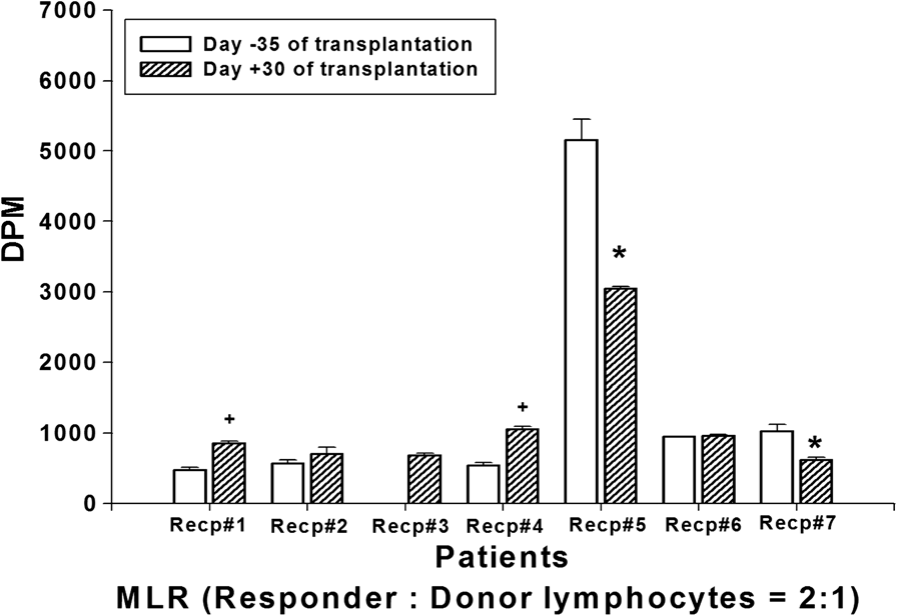
**Donor-Specific Lymphocyte Proliferation (mixed lymphocyte reaction).** Heparinized blood sample of recipient was collected before (day-35) and after (day + 30) the MSC-kidney transplantation. The recipients lymphocytes (responder) were co-cultured with irradiated donor lymphocytes (stimulator) for 4 days. Donor-specific recipients’ lymphocyte proliferation was determined by ^3^H-thymidine incorporation using a liquid scintillation counter (Beckman LS 6500; Beckman Instruments Inc., Fullerton, CA, USA). Proliferative response was represented by DPM. Plus signs indicate statistically significant induction of proliferative response (p < 0.05) compared to D-35 baseline activity. Asterisks indicate the statistical significance of decreased proliferative response compared to D-35 baseline activity (p < 0.05).

**Figure 3:**
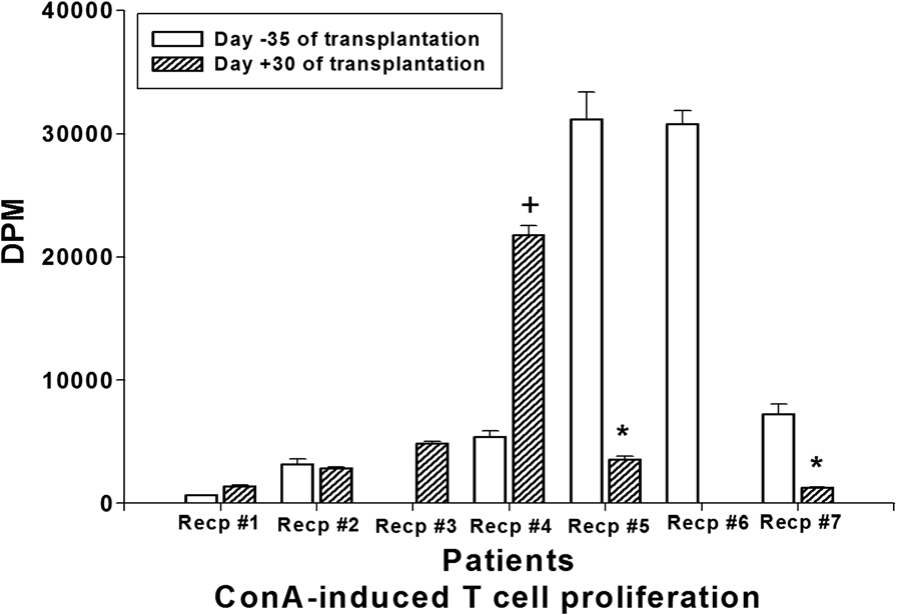
**Donor-Specific T cell Proliferation (mixed lymphocyte reaction).** Co-culture of recipient’s blood lymphocytes and irradiated donor lymphocytes was stimulated by the T cell mitogen, ConA. Same experimental procedure was performed as MLR for lymphocyte proliferation analysis. Proliferative response was represented by DPM. Plus signs indicate the statistically significant induction of proliferative response (p < 0.05) compared to D-35 baseline activity. Asterisks indicate the statistical significance of decreased proliferative response compared to D-35 baseline activity (p < 0.05).

Donor-specific proliferative response of recipient’s lymphocytes before (d −35) and after (d +30) the transplantation with MSC infusion was analyzed by MLR. Compared to the pre-transplantation, donor-specific lymphocyte proliferation was significantly inhibited in the patient 5 and 7 (Figure 2). On the contrary, in patient 1 and 4, increased lymphocyte proliferative activity was observed (Figure 2). Alteration of T cell proliferation induced by ConA, a T cell mitogen, was observed with similar pattern to the total lymphocyte proliferation (Figure 3). Unfortunately, for the ConA induced T cell proliferation, baseline data of the patient 3 and post-transplantation data of the patient 6 could not be obtained due to the lack of sample (Figure 3). The lymphocyte or T cell proliferative phenomenon was not correlated with inhibitory cytokine (IL-10) secretion or the induction of Treg presented by FoxP3 mRNA level (Table 3).

**Table 3:** Modulation of the immune parameters in the MSC-kidney transplantation recipients

**No**	**Age/Sex**	**Clinical outcome at 12 Month**	**Tregs**^**1**^	**Donor-specific lymphocyte proliferation**^**2**^	**Donor-specific T cell proliferation**^**3**^	**Cytokines**^**4**^
1	34/M	Borderline change	**↑↑**	**↑**	-	IL-10 at D + 7 IL-6 at D + 7
2	48/F	No AR	**↑↑**	-	-	IL-10 at D + 30
3	40/M	No AR	-	ND*	ND*	NC**
4	32/F	ACR	-	**↑**	**↑↑**	NC**
5	36/M	No AR	-	**↓↓**	**↓↓**	IL-6 at D + 7 IFN-γ at D + 30
6	36/F	Borderline change	-	-	-	
7	40/F	ACR	-	**↓**	**↓↓**	IFN-γ at D + 4

^1^Represented by the level of FoxP3mRNA : compared to baseline (D −35) control (Figure 1).

^2^Peripheral blood lymphocytes were co-cultured with donor cells (MLR) (Figure 2).

^3^ConA-induced T cell proliferation in the MLR system. (Figure 3).

^4^Cytokines their secretion was significantly increased compared to donor and baseline (D-35) level. (Figures 4, 5, 6).

*ND = Not detected: due to the lack of sample.

**NC = No changes.

↑ or ↓ = Increased or decreased values compared to baseline (D-35) control.

### Cytokine secretion

**Figure 4:**
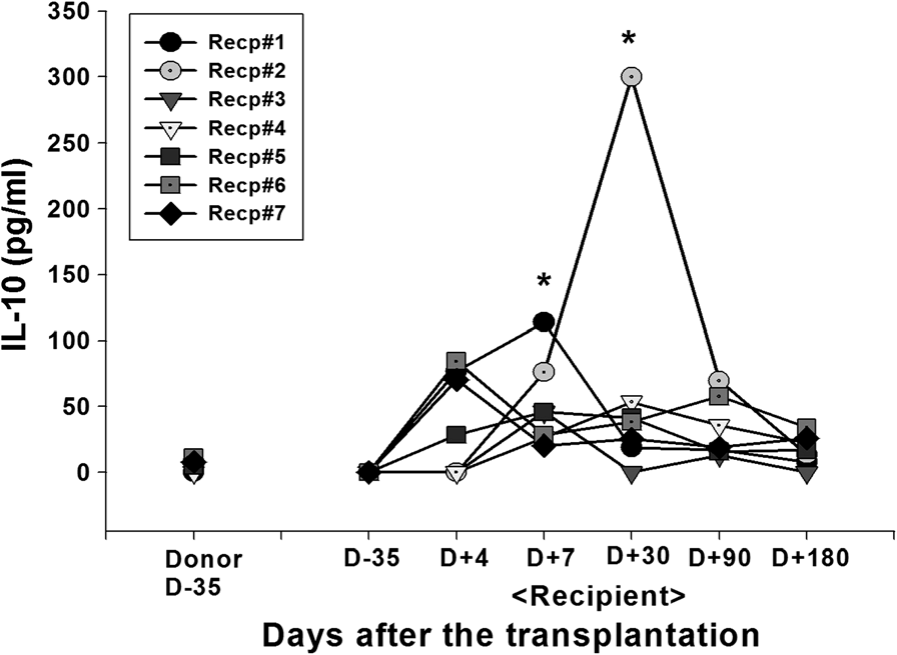
**IL-10 secretion.** Alteration in blood circulating concentrations of IL-10 was measured by ELISA. Heparinized blood sample of donor or recipient was collected before (day-35) and at various times after (day + 4, 7, 30, 90, 180) the MSC-kidney transplantation. Plasma was separated and stored at −70°C until the ELISA was performed using commercial kit from OptEIA™ (e-bioscience, San Jose, CA, USA). Asterisks indicate the statistical significance of cytokine secretion compared to D-35 baseline activity (p < 0.05).

**Figure 5:**
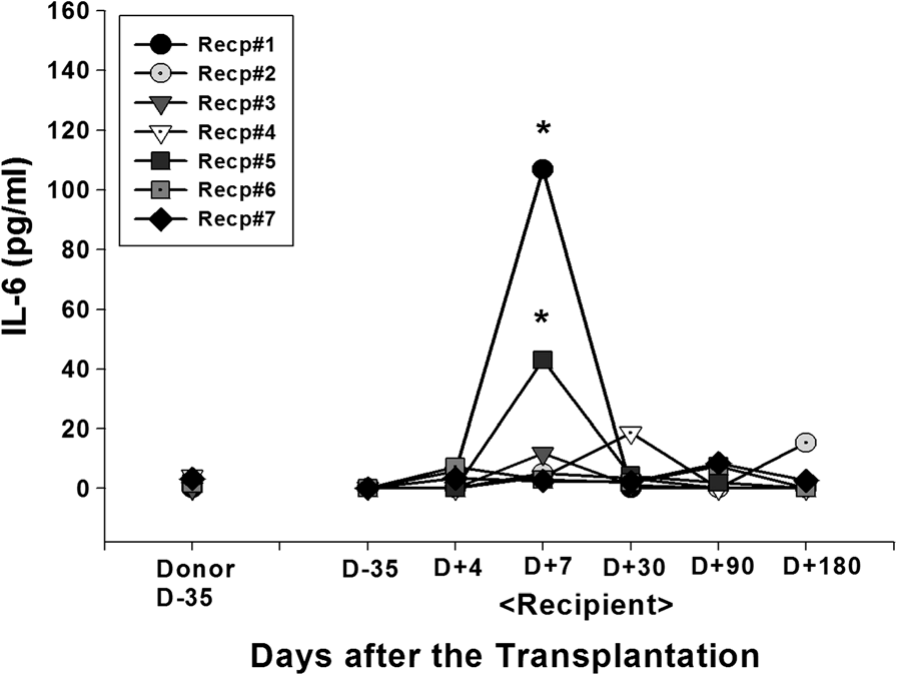
**IL-6 secretion.** Alteration in blood circulating concentrations of IL-6 was measured by ELISA. Heparinized blood sample of donor or recipient was collected before (day-35) and at various time after (day + 4, 7, 30, 90, 180) the MSC-kidney transplantation. Plasma was separated and stored at −70°C until the ELISA was performed using commercial kit from OptEIA™ (e-bioscience, San Jose, CA, USA). Asterisks indicate the statistical significance of cytokine secretion compared to D-35 baseline activity (p < 0.05).

**Figure 6:**
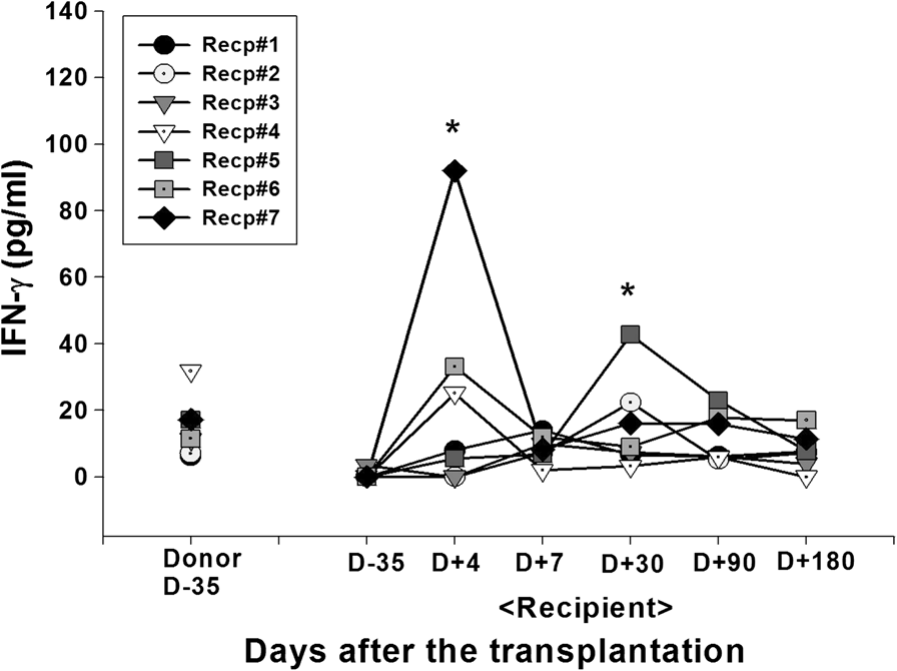
**IFN-γ secretion.** Alteration in blood circulating concentrations of IFN-γ was measured by ELISA. Heparinized blood sample of donor or recipient was collected before (day-35) and at various time after (day + 4, 7, 30, 90, 180) the MSC-kidney transplantation. Plasma was separated and stored at −70°C until the ELISA was performed using commercial kit from OptEIA™ (e-bioscience, San Jose, CA, USA). Asterisks indicate the statistical significance of cytokine secretion compared to D-35 baseline activity (p < 0.05).

Cytokine secretion into the blood was analyzed. Plasma level of IL-10, an immune inhibitory cytokine was significantly increased in the patient 1 at day 7 (0 vs 113.81 pg/ml for the pre vs post transplantation, respectively). In the patient 2, significant induction of IL-10 level was observed at day 30 after the transplantation (0 vs 299.91 pg/ml for the pre vs post transplantation, respectively) (Figure 4). Significant induction of IL-6 secretion at day 7 post-transplantation in patient 1 or 5 (0 vs 106.77 pg/ml or 42.89 pg/ml for patient 1 or 5, respectively) did not correlated with other immunological or clinical parameters (Figure 5). In the patient 7, early onset of IFN-γ secretion at day 4 (92.01 pg/ml) was observed (Figure 6). In the patients other than above mentioned, no significant alteration of cytokine level was observed.

## Discussion

MSCs exert immune-regulatory effects in vitro and in vivo, and this effect has lead to research involving MSCs in solid organ transplant tolerance induction models [7,16-18]. In this pilot clinical trial, feasibility along with the possibility of immune tolerance induction by intra-osseous injection of MSC in LDKT was observed. The intra-osseous route of MSC infusion was tried with expectation of better seeding efficiency of injected MSCs [19]. In our clinic, MSC injection into the recipient’s iliac bone at the time of kidney transplantation was safe and did not result in any sign of adverse effects. At the 12 month after the transplantation, in all seven MSC treated patients, engraftment failure had not occurred with the two cases of ACR which controlled well with SPT (Table 2). No differences in the clinical responses were observed between the non-MSC control and MSC-treated group (Additional file 1: Table S2).

Induction of inhibitory immune function was studied which may contribute to the reduction of the conventional immune-suppression after transplantation. The role of Treg in the transplantation-immunology has been reported and MSC are known to prime the Treg to induce the immune-suppressive effect [9,20]. The FoxP3 mRNA was measured in the patients before and various time after the MSC-kidney transplantation (Figure 1). Although statistical significance was not found, gradual increases of FoxP3 mRNA representing Treg was observed in the MSC group but not in the non-MSC control group (Additional file 3: Figure S2). Data suggested the possible role of injected MSC for inducing regulatory immunity in LDKT patients by one time intra-osseous injection with kidney transplantation simultaneously. With this data one could assume that one time injection of MSC may not enough to induce the clinically detectable responses. Donor-specific lymphocyte proliferation analysed by MLR and mitogen-induced T cell proliferation were increased in patients 1 and 4. Among these two patients, increased regulatory immune responses (Treg, IL-10 and IL-6 secretion) were observed only in patient 1 (Table 3). In the patient 4, but not in the patient 1, acute cellular rejection was occurred. On the other hand, significant reduction of donor-specific lymphocyte and mitogen-induced T cell proliferation were occured in patients 5 and 7 (Table 3). Interestingly, in those two patients, increased IFN-γ secretion was observed (Table 3). Originally, IFN-γ stimulates the effector lymphocytes rather than inhibits. However, the role of IFN-γ was also observed in the MSC and immunosuppressive drug treated murine heart transplantation group [20]. The authors [20] explain the immunosuppressant-mediated differential secretion of IFN-γ influenced the immunosuppressive effect of MSC. In this study, acute cellular rejection was observed in the patient 7 with significant induction of IFN-γ secretion at early time point (4 days after the transplantation).

Among the mechanisms of MSC-induced immune-suppression including the inhibition of effector T cell proliferation, regulatory T cell induction and immune-inhibitory cytokines like IL-10, TGF-b [8,9,21-23]. Our findings may contribute to reveal the possible relationship between the clinical outcomes (transplant rejection) and the inhibitory immune responses in MSC-kidney transplant patients. However, to insure the findings, it may necessary to compare the data from non-MSC control group. More cases should be analyzed to conclude for the role of MSC in immune regulation to transplanted organ. Further clinical study is planned to solve these problems.

## Competing interests

Authors do not have any conflict of interest.

Dr. Kim, HyunSoo from FCB Pharmicell CO. did provide the clinical grade MSCs which were prepared in their KGMP facility. Clinical grade MSCs were given with no commercial relevance connected with FCB Pharmicell.

## Author’s contributions

HL and JP prepared the protocols of clinical trial and immune monitoring, perform the experiments, analyzed the data and wrote the manuscript as first authors. SL and SB also carried out the experiments and helped to analyze the data. HK participated in the design of the study and generation of clinical grade MSCs. As a corresponding author, SK designed and supervised the whole project for execution. All authors approved the final manuscript.

## Supplementary Material

Additional file 1: Table S1 Characteristics of the patients including non-MSC control group. **Table S2.** Patients’ profiles of the non-MSC control group. Click here for file

Additional file 2: Figure S1 Level of urine FoxP3 mRNA in the LDKT with non-MSC control group. Click here for file

Additional file 3: Figure S2 Level of urine FoxP3 mRNA in the LDKT with non-MSC control group. Click here for file
