# Supplementary material for: Intra-osseous injection of donor mesenchymal stem cell (MSC) into the bone marrow in living donor kidney transplantation; a pilot study
Source: J Transl Med. 2013 Apr 11;11:96. doi: 10.1186/1479-5876-11-96 (PMC3630056; doi:10.1186/1479-5876-11-96)
Supplement: Additional file 1: Table S1 — Characteristics of the patients including non-MSC control group. Table S2. Patients’ profiles of the non-MSC control group. [file 1479-5876-11-96-S1.doc]

**Supplement Table 1. Characteristics of the patients**

|  |  | | **MSC group (n=7)** | **Non-MSC Control group(n=4)** |
| --- | --- | --- | --- | --- |
| **Recipient** | **Age (year)** | | **36.0 (32~48)** | **36 (21~46)** |
|  | **Sex (M : F)** | | **3 : 4** | **2 : 2** |
|  | **Etiology** | |  |  |
|  |  | **Unknown** | **4** | **0** |
|  |  | **Diabetes** | **0** | **0** |
|  |  | **Hypertension** | **0** | **1** |
|  |  | **IgA nephropathy** | **2** | **2** |
|  |  | **GN** | **1** | **1** |
|  | **Underlying DM / HTN** | | **2 / 3** | **0 / 2** |
| **Donor** | **Age (year)** | | **38 (29~48)** | **44.5 (41~50)** |
|  | **Sex (M:F)** | | **6 : 1** | **2 : 2** |
|  | **Relation (Related /Unrelated)** | | **7 : 0** | **2 : 2** |
|  | **HLA mismatch (0/1/2/3/4)** | | **1 / 0/ 3 / 1 / 2** | **0 /0 / 2 / 2 / 0** |
|  | **DR mismtach (0/1 /2)** | | **2 /5 /0** | **1 /3** |

GN, glomerulonephritis; DM, Dibetes Mellitus; HTN, hypertension

**Supplement Table 2. Patients’ profiles of the non-MSC control group.**

**Control (non-MSC group; n=4)**

| No | Age/Sex | Etiology | Donor | | HLA  mismatch | Rejection within 12Mo | Protocol Bx  at 12 Mo |
| --- | --- | --- | --- | --- | --- | --- | --- |
|  |  |  | Relation | Age/Sex |  |  |  |
| 1 | 46/M | IgA nephropathy | Wife | 41/F | 3 | AR | No AR |
| 2 | 45/F | HTN | Husband | 46/M | 2 | - | - |
| 3 | 28/F | IgA nephropathy | Father | 50/M | 2 | - | - |
| 4 | 21/M | GN | Mother | 43/F | 3 | - | - |

GN, glomerulonephritis; HTN, hypertension; AR, acute rejection.
