# Supplementary figures and images for: Intra-osseous injection of donor mesenchymal stem cell (MSC) into the bone marrow in living donor kidney transplantation; a pilot study
Source: J Transl Med. 2013 Apr 11;11:96. doi: 10.1186/1479-5876-11-96 (PMC3630056; doi:10.1186/1479-5876-11-96)

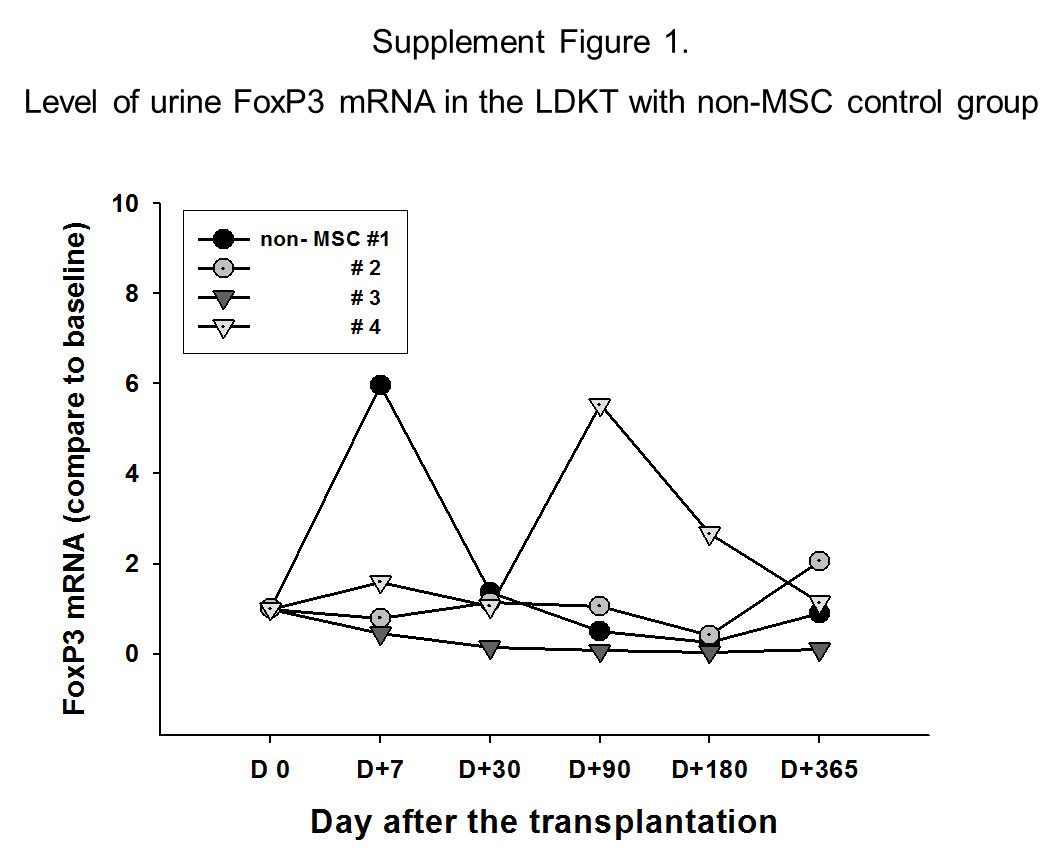

Supplement: Additional file 2: Figure S1 — Level of urine FoxP3 mRNA in the LDKT with non-MSC control group. [file 1479-5876-11-96-S2.tif]

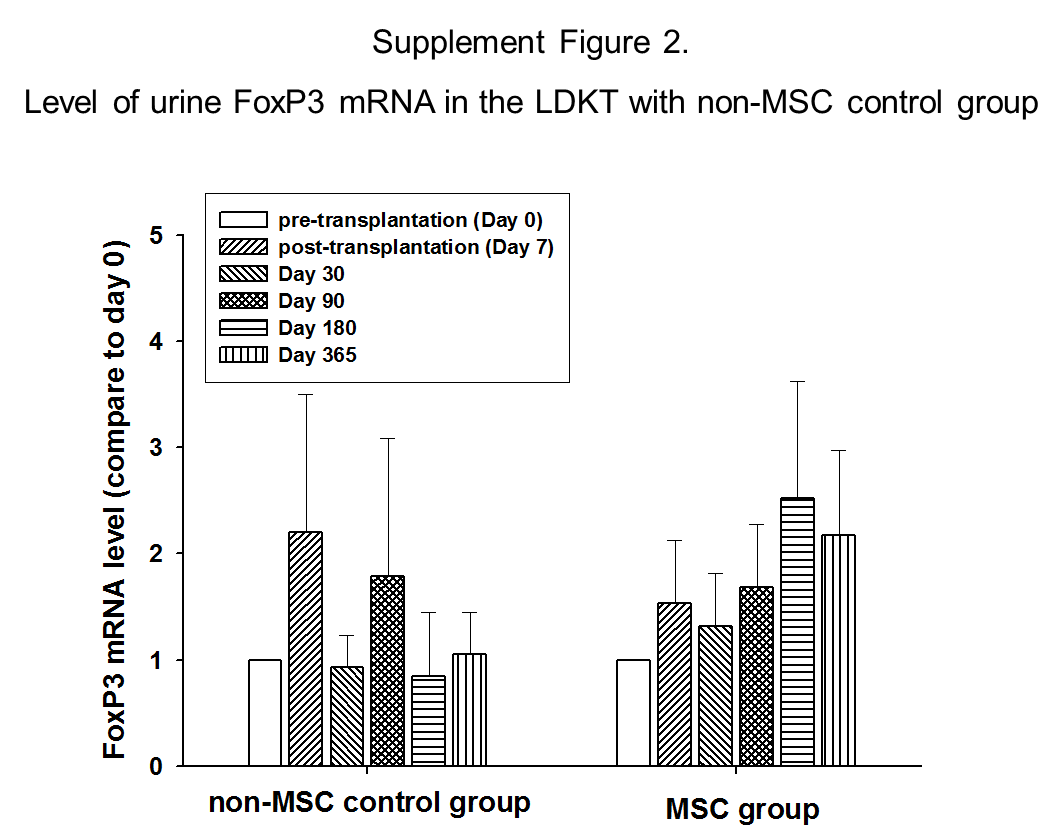

Supplement: Additional file 3: Figure S2 — Level of urine FoxP3 mRNA in the LDKT with non-MSC control group. [file 1479-5876-11-96-S3.tif]
